# Sensor-Based Passive Remote Monitoring and Discordant Values: Qualitative Study of the Experiences of Low-Income Immigrant Elders in the United States

**DOI:** 10.2196/11516

**Published:** 2019-03-25

**Authors:** Clara Berridge, Keith T Chan, Youngjun Choi

**Affiliations:** 1 University of Washington Seattle, WA United States; 2 University at Albany - State University of New York Albany, NY United States

**Keywords:** immigrants, ubiquitous sensing, acculturation, passive monitoring, independent living, family caregiving, culturally appropriate technology

## Abstract

**Background:**

Remote monitoring technologies are positioned to mitigate the problem of a dwindling care workforce and disparities in access to care for the growing older immigrant population in the United States. To achieve these ends, designers and providers need to understand how these supports can be best provided in the context of various sociocultural environments that shape older adults’ expectations and care relationships, yet few studies have examined how the same remote monitoring technologies may produce different effects and uses depending on what population is using them in a particular context.

**Objective:**

This study aimed to examine the experiences and insights of low-income, immigrant senior residents, family contacts, and staff of housing that offered a sensor-based passive monitoring system designed to track changes in movement around the home and trigger alerts for caregivers. The senior housing organization had been offering the QuietCare sensor system to its residents for 6 years at the time of the study. We are interested in adoption and discontinuation decisions and use over time, rather than projected acceptance. Our research question is how do cultural differences influence use and experiences with this remote monitoring technology? The study does not draw generalizable conclusions about how cultural groups interact with a given technology, but rather, it examines how values are made visible in elder care technology interactions.

**Methods:**

A total of 41 participants (residents, family, and staff) from 6 large senior housing independent living apartment buildings were interviewed. Interviews were conducted in English and Korean with these participants who collectively had immigrated to the United States from 10 countries.

**Results:**

The reactions of immigrant older adults to the passive monitoring system reveal that this tool offered to them was often mismatched with their values, needs, and expectations. Asian elders accepted the intervention social workers offered largely to appease them, but unlike their US-born counterparts, they adopted reluctantly without hope that it would ameliorate their situation. Asian immigrants discontinued use at the highest rate of all residents, and intergenerational family cultural conflict contributed to this termination. Social workers reported that none of the large population of Russian-speaking residents agreed to use QuietCare. Bilingual and bicultural social workers played significant roles as cultural navigators in the promotion of QuietCare to residents.

**Conclusions:**

This research into the interactions of culturally diverse people with the same monitoring technology reveals the significant role that social values and context play in shaping how people and families interact with and experience elder care interventions. If technology-based care services are to reach their full potential, it will be important to identify the ways in which cultural values produce different uses and responses to technologies intended to help older adults live independently.

## Introduction

### Background

The availability of long-term care provided by family and professional caregivers is projected to fall short of growing demand in the United States [1]. Technology-based care services have been presented as a potentially elegant solution to the issue of insufficient numbers of elder care workers and family caregivers as well as a means to address disparities in access to care for the growing elder immigrant population in the United States. The most well-diffused technology to support aging in place is sensor-based passive remote monitoring [2]. Sensor systems are used to monitor the activity and movement within the living space of older adults with the goal of detecting deviations from one’s normal routine and long periods of inactivity that may indicate an emergency. These systems are passive in that they require no action on the part of the older adult, unlike “active” personal emergency response systems (PERS) requiring that a button is pushed to actively call for help. Older adults often want to avoid making their adult children’s busy lives more complicated because of their health problems and do not want their children to be overly concerned about them [3]. Passive remote monitoring has the potential to support this goal.

### Where Cultural Values and Monitoring Technologies Intersect

The notion that technology is value-neutral and asocial is disputed by research that reveals how socially embedded technologies are—from their conceptualization through performance in real-world situations [4-6]. In the case of gerontechnology, technology developers—who are always socially and culturally positioned—are designing for people who are differently socially and culturally positioned. A device designed to support independent living is typically conceptualized and engineered by relatively young men who envisage their user as someone who is old, possibly with chronic conditions, and who prioritizes living at home [7]. Technology is also socially embedded because devices have particular goals that necessarily prioritize values: for example, risk management may be prioritized over privacy or social interaction. This social science insight that technology is social and embedded with values challenges the assumption of a neutral relationship between health technology, thereby challenging neutrality with regard to culture in which values are formed and acted upon [8]. The conceptualization of remote monitoring as both a technical and always social practice makes evident the need for research on the associated social and cultural meanings.

A number of studies have examined racial and ethnic differences in the use of assistive devices for mobility in the United States and have found that older adults of color have higher utilization than non-Hispanic whites [9-11]. The higher use appears to be driven by an acknowledged underlying need to compensate for the loss of functioning [9,10] and prevent falls [12] but can be impacted by a lack of financial resources [11,13] or difficulties in access [13,14].

Understanding cultural context is critical for understanding the implementation and use of information technology [15]. Cultural factors can influence how information technologies such as remote monitoring may produce different effects. In an ethnographic study of a personal emergency response system in Sweden and the United States, acceptance and use by participants were affected by how they connected the technology to their respective health care systems [16]. Swedish older adults received the emergency response system as part of a publicly provided home care service through their care workers and regarded access to current technologies through the welfare system as a right. In contrast, older American adults understood the service as a good they purchase as customers. American older adults expressed more hesitations about the technology-based service that made them less likely than their Swedish counterparts to use it [16]. A comparative study of home monitoring systems with Korean and Korean American older adults similarly revealed that cultural factors impact acceptability. The study identified impactful cultural factors of independent living because of immigration and loosened filial tradition, satisfaction with health care services, and technology capacity building by the government [17]. Both of these studies underscore the need to examine cultural contextual factors in research on acceptance and experiences with monitoring technologies by older adults.

Examining possible cultural differences in the use of elder care technologies is also important in the context of a growing immigrant population in the United States and the need for new solutions to address the cultural gaps in care services for these aging populations. In this study, we examine the experiences of immigrant, senior housing residents with a sensor-based passive monitoring system offered to them on a voluntary basis by a subsidized senior housing organization. The sample population is uniquely diverse, with senior housing resident participants from 10 countries. This research into the interactions of culturally diverse people with the same monitoring technology reveals the significant role that social values and context play in shaping how people and families interact with and experience technology-based elder care interventions.

### Acculturation by Elder Immigrants in the United States

The US immigrant older adult population jumped from 2.7 million in 1990 to 4.6 million in 2010, an increase of almost 70% within 20 years [18]. According to 2010 Census estimates, immigrant elders comprise 11.9% of all adults aged 65 years and older in the United States. This demographic shift reflects large-scale changes in patterns of migration, with Asians becoming one of the largest pan-ethnic immigrant groups since mass migration from this region began in 1965. Migration from other countries such as former Soviet republics has also reflected changes in geopolitics since the fall of the Soviet Union, with the population of Russian speakers more than tripling from 1990 to 2010 [19].

For immigrant studies, acculturation has been often operationalized as the extent to which immigrants adopt host cultures [20]; however, researchers are increasingly understanding acculturation as a blending of different cultural values into immigrants’ adaptation process rather than as a linearized process toward acceptance [21-23]. For example, although Asian elder immigrants in the United States may retain filial piety as a core value that supports intergenerational care [24,25], they also modify its application to new cultural realities in the United States where independence is emphasized [16,20]. Studies have shown a trend among Korean, Chinese, and Indian elder immigrant groups toward a preference for more independence from their adult children [20,26], describing how these groups may associate filial piety with emotional social support rather than direct care [16,26].

With family care expectations in flux, help-seeking behavior outside of family systems is an important area of focus. For one, research indicates that the complexity of navigating value systems, language, customs, lifestyle, and the Westernization of their children prevents Chinese and Korean immigrants from seeking help from English-speaking service providers [27,28]. A filial piety framework has been used to explain barriers to seeking formal elder care services, yet this principle does not fully address the dynamics of elder health and long-term care services when they are accessed by immigrants. Confucianism encompasses other principles seldom employed in health and social services research in the United States that may promote understanding of immigrant elders’ behaviors. One such value that we employ in this study is the primacy of a benevolent, trusting relationship between a governing authority and subject (the literal translation is king and citizen) that is rooted in the principle of paternalism, which can be thought of as the extension of the family to the larger social whole [24,29]. Aspects of Confucian value systems are likely relevant along with filial piety, and these values may be pieces that help compose a stronger understanding of Asian immigrants’ interactions with extrafamilial health and home care services.

Much hope has been placed in the use of technologies to remotely monitor health and safety in ways that are more broadly accessible and acceptable. We take as a starting point that new practices developed around technology for elder care are social practices and that technologies are embedded with values. Providers cannot assume that all users hold the same values that shape their experiences with a given technology. Applying an understanding of acculturation as a process can provide insights into immigrant older adults’ interactions with remote monitoring technologies. Immigrant elders’ adaptation of new technology can be understood as a cultural integration process in which they develop their own meaning of care technologies while calibrating their own cultural values with those of their host culture. Examining interactions with technology through the lens of immigrant older adults can lead to greater and more successful utilization of these innovations in everyday life.

## Methods

### The Technology Intervention and Field Site

The field site is 6 subsidized independent living residential buildings owned by a large senior service and housing not-for-profit, mission-driven organization in a US metropolitan city. The housing organization had been offering QuietCare to its residents for a highly subsidized rate of US $5 to 15 per month for 6 years at the start of this study, so implementation issues typical for new technologies had long been worked out. QuietCare comprised 5 interconnected sensors placed in predetermined locations throughout the resident’s apartment (bathroom door, bedroom door, apartment door, refrigerator, and environmental temperature sensors). Family members and social workers can access information about changes in movement from the individual’s norm that may indicate a problem. The system also triggers alerts when certain sensors detect no movement (eg, if there is no motion through the bedroom door in the morning, if someone does not come out of the bathroom in a given time, or when no movement in the home is detected for a period of time). The system is connected to a telecare call center that first tries to reach the older adult, followed by family emergency contacts, and in some cases, the emergency medical service (EMS) is contacted when no one can be reached. The study received ethical approval from The Committee for Protection of Human Subjects, University of California, Berkeley.

### Participants and Recruitment

In-depth semistructured interviews were conducted in English and Korean with elder residents, family members, and staff. As depicted in Table 1, a total of 41 participants were interviewed. Family members and residents were interviewed once, and social work staff were interviewed twice. All participants were US citizens, and all residents had multiple chronic conditions. Residents had incomes less than $36,120; a total of 5 participants had incomes less than $18,050. The majority of the residents had completed high school.

There were a total of 23 current users of Quietcare, of which 8 were not recruited because of serious health issues and dementia (n=4) and because they did not speak English or Korean (n=4). Of the remaining 15 current users, all were invited and agreed to participate in the study. We invited each of the 8 residents who had discontinued the system in the past 12 months and 3 refused to be interviewed: 2 were Chinese American and 1 was Korean American. The resident participants included individuals born in the United States, Poland, Czech Republic, Puerto Rico, Peru, Malaysia, Japan, China, and South Korea. Multilingual social workers were able to explain the reasons for discontinuation of the system among those who refused to be interviewed and could speak about resident use and discontinuation over a 6-year period since QuietCare had been available.

**Table 1:** Study participants.

Participant group	Number of participants interviewed	Number of foreign-born participants
Residents	20 (including 5 who had discontinued the QuietCare system)	11
Emergency contacts	11 (2 who had discontinued)	4
Staff^a^	10	7
Total	41	22

^a^All but 2 staff were interviewed twice; 2 supervisors were interviewed only once because they did not have regular interactions with residents who use QuietCare, and all their knowledge and experience with the system could be discussed in a single interview.

Family members were recruited with the permission of participating residents. Some older adults did not want to burden their family member with the request, and 11 family members who served as emergency contacts were invited and agreed to be interviewed. These were daughters, sons, granddaughters, sisters, and 1 long-term home aide born in China, Korea, Guyana, India, and the United States. All technology, housing, and social work staff who had contact with the technology participated. The 10 staff members included 3 US- and 7 foreign-born participants from Russia, China, Israel, India, and Korea. Staff members were multilingual. Language and cultural congruence between staff and the residents was often achieved. All social work staff were women (n=6), and all housing and technology staff were men (n=4).

### Data Collection and Analysis

The first author conducted all but 5 interviews and attended all interviews. The Korean interviews were coconducted by an interviewer who was fluent in Korean and English. The interviews were semistructured. Social work staff were interviewed twice: once before interviewing residents and family members and once after. This provided the time needed for an in-depth discussion about their experiences promoting the technology, the opportunity to triangulate the interviews, and the opportunity to follow up with social workers on cultural differences they perceived were at play in residents’ experiences with the technology. Residents and family members were asked about how they made decisions about adoption (and when relevant, discontinuation) and about their experiences with the system. Residents were invited to tell their story of how they came to live in the senior housing building, and they discussed their immigrant histories and family situations. Social workers were asked about their observations of adoption and system discontinuation decisions and processes, about their own experience of using the system and daily interactions with the sensor-generated data, and about cultural differences in adoption decisions and experiences of their residents. Some had worked at the organization since before QuietCare was offered, so they were able to speak about the 6 years of experience working with residents and QuietCare.

Audio-recorded interviews were transcribed verbatim. The 5 interviews conducted in Korean were transcribed in Korean and then translated into English. The first author conducted the analysis and discussed cultural translations and clarified meanings with the researcher who conducted the Korean interviews. All interviews were coded beyond the point of conceptual saturation to reduce the potential for coder bias. Transcripts were imported into Dedoose (University of California, Los Angeles), which is a software that facilitates the management of qualitative data.

A multistep coding process of grounded theory was used, beginning with open coding and followed by axial coding in which categories of codes were connected so that dominant themes could emerge to produce an explanation of patterns in the ideas heard across interviews [30,31]. Examples of themes are “fear of burdening adult children” and “appeasing social worker.” As part of this constant comparison method, themes were compared and contrasted across individual interviews [31]. The interview guides and a detailed description of the study participants and analytic methods can be accessed in the study by Berridge et al [32].

## Results

### Values Made Visible

The reactions of immigrant older adults to the passive monitoring system reveal a tension between their values, needs, and expectations on the one hand and the values embedded in the technology on the other. Here, we describe themes of cultural difference in response to the sensor system: differing expectations for how one should receive care and support and reactions of hope versus skepticism that the sensors will help, different reactions to the involvement of family members as emergency contacts, and experiences of intergenerational family cultural conflict. We also provide insights into the work of the multilingual and bicultural social workers as cultural navigators, including linguistic and cultural barriers to successful marketing and service delivery on the part of the technology company.

Russian social workers explained that none of the more than 200 Russian-born residents saw enough added value above the active PERS to adopt passive monitoring, citing that most of them had Medicaid, which covers PERS. Social workers observed that their Russian clients felt that they should be provided hands-on support and favored “concrete care” over remote monitoring. One social worker explained that Russian residents ask the rhetorical question, “Why should I depend on a piece of plastic for my life?” Another described what she called “socialist thinking,” noting that Russian elders grew up in a country where they received free education. She recalled how a resident had approached her in anger because a fellow resident was in need of help but was only offered the passive monitoring system:

She came in and she was very angry with me. She said why doesn’t Sarah have a home aide? She deserves a home aide. Why hasn’t [the housing organization] provided her one?

The social worker recalled having a difficult time explaining to this Russian-born resident that her neighbor did not have a home aide, despite her need, because she was not eligible for Medicaid and could not afford to pay out of pocket. These residents preferred in-home human support and expected that it be accessible.

Contrasting values also surfaced in the response of many Chinese- and Korean-born residents to one of the primary purposes of passive monitoring. The assumption that an older adult wants an early intervention if she is severely ill was challenged by some of these residents who stated that “When the time comes, I am ready.” They expressed a desire to avoid prolonging a severely impaired life and to pass away when the time came. This relationship with death involves a high degree of acceptance and differs from a desire to intervene to the end, characteristic of the US health care system [33]. Along similar lines, optimism about the efficacy of passive monitoring as a safety feature was shown to be a cultural commitment. Social workers reported that English-speaking and US-born residents adopted the technology because they had a fall history and genuinely hoped it would help them, whereas Asian-born residents were less optimistic that it would help them, accepting the monitoring system only if they had no one to depend on or little family contact.

### Burdening and Intergenerational Family Cultural Conflict

A common theme among Asian elder immigrants was their experience of intergenerational family cultural conflict, which was most often reflected in their disappointment over being asked by their children to live in a senior housing building. Compared with their US-born residents, Chinese- and Korean-born residents who adopted the sensor system were particularly concerned about troubling or burdening their children. In combination with the family cultural conflict surrounding shifting norms, frequent false alerts of passive monitoring created a tension that led Korean and Chinese American residents to discontinue the system at the highest rate compared with all other users. Social workers attributed this in large part to role conflict. One explained:

So they have the image that they do not want to bother their children. I think because especially this Asian group now, they have experienced so many wars...they have so many things suffering feeling unfairly treated for their whole life but they will not say it. Even at this time that they do not think a parent mother should bother the adult children, but rather, they should protect and take good care of the children and when they grow older they don’t want to become the burden.

This feeling that the false alarms caused them to be a burden on their children was particularly disturbing to women who were accustomed to taking care of their younger family members and did not wish to become the care recipient in the family while living away from the family in senior housing. Social workers reported that it was not the family members who were bothered by the false alerts, but rather, the older adult:

It is not the children who are complaining: “Why did QuietCare call during the night when there was a false alarm?” Or “Why did they call me when I am busy during the day?” The children of course will feel bad, but they do not say, “Okay, cut it off.” They do not say that. It is the senior: “Oh please, please.” They even beg me, “Please let me go. I did not want to bother them [their adult children].”

The experience of causing burden rather than alleviating it in the context of new norms of living apart contributed to the high rate of discontinuation among Asian immigrants. These residents concluded that the technology was useless and burdened their family members.

### The Role of the Cultural Navigator

Social workers who were multilingual and able to relate to residents who had emigrated from the same country or region as they had served as buffers to the outside world that frequently involved discrimination and poor treatment. One Russian-speaking social worker explained:

But the environment – I mean social workers speak Russian the social worker speaks Chinese – residents feel less stress with them, but in the real life outside world it is stressful.

The relationships immigrant residents had with their social workers were important because they were some of the few people who patiently sought to understand them and their concerns and because they represented a safe space in the context of what could be a hostile outside environment. These relationships served as leverage during decision making about remote monitoring adoption, which the social workers were responsible for achieving.

All social workers noted that their relationship with their clients was key to getting them to adopt the technology. A Chinese-speaking social worker explained:

The Asian group, I don’t think they are so much interested in it [passive monitoring]. I have to say to you, I think they accept it because I promoted it...I think there still is one or two in the program because I promoted it. I know they don’t like it. But then it’s only five dollars. To “let’s not give [social worker’s name] a hard time” or something. Comparing cultures, if I can use the authority even though I'm not an authorized person, but because “oh, staff say this and even though I prefer not to, okay let me have it.” If they have a choice to make their decision, they would not want to have it but they have it because I promoted it.

Chinese and Korean residents may have accepted monitoring because their social workers wanted them to have it. Their perceived relationship with their social workers was important to them for reasons that are unique to their experiences as immigrants who placed a high value in their formal and informal networks, as part of their everyday transactions.

As immigrants, social workers also drew on their experiences and skills navigating cultural difference to promote the technology. One explained:

Culturally there’s a big difference. So when we approach difference language groups, it’s different. I have to switch my channel [laughs]. It doesn’t mean respect or disrespect which group but it is a way of presenting something to a certain language group. For example, for certain groups you cannot be too passive, you have to explain and then you have to take the lead.

Social workers who were immigrants were aware of the work they did to “switch channels” while interacting with residents from a variety of backgrounds who had different expectations of how they should be treated and how services are offered. When discussing the passive monitoring system, they delicately navigated word choice so as not to disturb or put off residents. A social worker described the difficulty they had discovering a word that would not convey intrusiveness:

We are very, very cautious about the words we use. We cannot say there’s an image, we cannot say we are monitoring, we cannot say we are watching you, but then eventually what’s the word I can use [laughs]?! We’re very careful. We try not to use monitor, we try not to use image, then all of a sudden, what to tell them? [laughs].

Eventually, through trial and error, social workers found that residents understood the example of a “sweeping motion” the buildings’ sensors detect to open and close doors as people step into the entries. Still, they encountered residents who did not trust that the sensor system was not a camera. A social worker noted:

In the beginning [the residents] just don’t believe us...but I still have to be very careful about monitoring and picture. We definitely don’t say that.

Due to their cultural positioning and close relationships with residents, social workers had valuable insights about barriers to adoption among immigrant residents. Social workers organized information meetings to advertise the sensor system, holding both single language and simultaneously translated meetings in Korean, Chinese, Russian, and English, but they were unable to bridge all language barriers. A language line was used by the telecare call centers, but all signal alerts that went through the telecare center were responded to in English. The operator would call using English to learn first if the resident needed help before dialing in the appropriate language line. For residents who did not speak English, having the telecare operator call in response to a signal and greet them in English was troubling. Through an initial attempted conversation, the operator would realize that the person was there but that they do not speak English. At that point, they would either look up the resident’s preferred language listed on that resident’s file or hear the resident repeating their preferred language in English and connect a translator on the phone through a language line. The reason the initial call was not made in the preferred language appeared to be a combination of timing and cost savings: the language line was an expense when used, so if a resident did not respond to a call or was not home, that time would be wasted money. Sometimes family members were conferenced in to translate, as an Indian-American son of a Hindi-speaking resident explained:

They will call her first and then they’ll call us and say we have her on the other line and can you help us talk to her?

This delayed language access presented a problem for some residents because it was alarming and confusing. A Russian-speaking social worker stated that the Russian residents were scared when a voice comes on in English:

They scared for this. A lot of people. Even people who live here a long time, like let’s say like 20 years.

A Chinese-speaking social worker described how she tried to prepare residents for this:

When I introduce it to the non-English speaking group, I will tell them they are supposed to be provided language. But just in case, you have to at least learn one word: “Chinese, Chinese, Chinese!” I would tell them. If unfortunately, an emergency happens, then you need help but you don’t speak English, then this is the word you have to learn.

A Chinese resident said that this is less easy in practice. “When stressed,” she explained, “you forget everything so you speak Chinese.” Residents described how being in an emergency, afraid, and possibly in shock could make one sensitive to incomprehensible words.

Language barriers also emerged in in-person interactions over the sensor system when social workers were unable to mediate. A Korean-speaking resident had discontinued the sensors after a false alert triggered a call to EMS and they broke her door to enter, not knowing she was not home. She explained, as she became visibly upset over an incident that occurred 2 years prior:

So the super came and he came for this, and saw the door and said I have to compensate for the door. So I have to pay for the door. So I haven’t even done anything wrong, I wondered why I should pay for it, but you know, I couldn’t say anything back in Korean, in English, so I haven’t said anything to the super. The super who manages this apartment said that since I broke the door, I should, we should, pay for it. So I was like, I haven’t done anything wrong, I have no sin, the firefighters broke it like this and they’re asking me to pay, so I haven’t said anything and I couldn’t speak English and I just left.

False alerts that caused EMS to arrive was a source of intense embarrassment for all residents who experienced it. The added language barrier when dealing with the consequences contributed to an unforgettable negative experience with the technology.

Another issue social workers drew our attention to is what they called a lack of cultural awareness and humility expressed by the technology company and their representatives. For one, the name of the product is not translatable into Korean, so an extra step is required to educate residents about what the system is. A social worker explained:

I have to teach clients to pronounce QuietCare in English so people know what they’re talking about. I write it out for them. The company does not have flyers in other languages.

She also described culturally insensitive company representatives and lack of diverse representation on product media:

Two white ladies from QuietCare came to present. They were far from sensitive and don’t understand their clients.

Social workers felt that this lack of cultural humility and awareness may have served as a deterrent to some residents. Social workers also noted how images of Asian elders were not included on any of the company-provided materials or website and that human diversity in marketing is limited to the depiction of African Americans and Latinos.

## Discussion

### Principal Findings

This study contributes to the limited literature on cultural specificity and remote monitoring technology. Our sample and qualitative methods drew out expectations and values at play that shed light on decisions made by Russian-speaking elders to decline the intervention and immigrant Asian elders’ reluctant adoption and high discontinuation rate. Our findings are consistent with previous research that illustrates how expectations rooted in cultural values and specific health care system contexts influence the embrace, reluctant use, or rejection of technologies [24,25]. Here, we highlight and discuss key themes that emerged at the point of acceptance or rejection of the QuietCare system, points of discontinuation, and the role of intergenerational family cultural conflict and the social worker as a cultural broker.

The first theme presented was the clash between Russian-speaking residents’ expectations for support and the support offered to them by the technological intervention. None of the more than 200 Russian-speaking residents accepted remote monitoring. Social workers attributed this to the desire for hands-on care (“Why should I depend on a piece of plastic for my life?”) and the expectations that this should be provided to them based on sociopolitical norms of publicly provided services. They understood this rejection of the intervention they were offering among this group of residents to be grounded in “socialist thinking” and expectations for support in old age that represented “concrete care.”

Chinese and Korean residents responded altogether differently than Russian-speaking residents and most US-born residents. Traditional Confucian values dictate that there is a filial obligation to provide for one’s elders in later life. With the breakdown of the traditional family network as a consequence of migration and modernization, Asian elders were in a quandary in regard to who they can turn to for support. They were aware of the demands of their own children, yet they were not fully acculturated to the expectation of service from outside their families. Social workers observed, and QuietCare users and former users confirmed, that residents from Asian countries generally did not believe the technology would be useful to them, and those who accepted it did so only when they lacked frequent family contact or alternative supports. These residents lacked hope that the intervention would help them and adopted the intervention reluctantly after social workers pressure them.

Social workers contrasted their Russian-speaking client’s tendency toward hard questioning about the technology with Asian resident’s reluctance to ask questions. Bicultural social workers expressed this in terms of passivity and the desire not to upset them. Recall the Chinese-born social worker who explained, “I think they accept it because I promoted,” an observation echoed by the Korean-born social worker. We can think of this dynamic of reluctant adoption so as not to make waves in the context of the Confucian values of filial piety and governing authority and subject and also a way to reciprocate in their relationships with those who help them, namely, the social workers they work with.

The principle of governing authority and subject emphasizes the fulfillment of loyalty to honorable leaders [29] and obeying a superior based on mutual trust and benevolence [24]. Social workers were the access point for needed public services, including the Department of Housing and Urban Development-subsidized housing in which they worked and residents lived. In this position, social workers represent government and authority. Findings indicate that the social workers’ promotion of the sensor system was successful despite the lack of enthusiasm by residents because these residents showed mutual respect for their social workers in a form of obeying organizational expectations embedded in the value of governing authority and subject, in return for the services provided. Bilingual and bicultural social workers were attentive to norms of a hierarchical age order and provided language support to residents, satisfying Asian older adults’ needs to be respected with proper filial traditions. Resident participants effusively expressed gratitude for their social workers during the interviews because they experienced the outside community as hostile or discriminatory in contrast to the safety and support they experienced in the residence buildings that social workers oversaw. The older adults’ acceptance of monitoring can be understood as behaviors of role fulfillment and gratitude for the social workers who provide emotional and material support. Older adults appeared to suppress their personal desire to reject the intervention in favor of promoting peace by obeying their social workers who represent a governing authority and not “giving social workers a hard time” by declining the recommendation to adopt.

Given this reluctant adoption reported by and about Asian immigrant residents, it is no surprise that this group would have the highest discontinuation rate, but we find that intergenerational family cultural conflict is also contributing to the decision to terminate the use of QuietCare. When false alarms caused unnecessary interruptions for their children at work or long drives to check on them, these residents were more likely to immediately terminate monitoring services out of concern that it was bothering their children. Social workers pointed out that it was not the children who complained about false alerts but the older adults. Korean and Chinese elders’ strong concerns could be situated within the cultural value of prioritizing the best interest and success of their children, even perhaps over their own well-being. This can also be explained as “saving face” behaviors that allow one to avoid any embarrassing situation. Korean and Chinese residents may be very sensitive to self-disclosure issues, considering the false alert may cause a threat to maintaining their self-esteem. These residents expressed to their social workers fears of burdening their children and often concealed feelings of sadness about living in a senior housing building to avoid burdening their children. These findings indicate that when implementing remote monitoring interventions, it is important to be attentive to possible differences regarding sensitivity to self-disclosure.

The findings also highlight the ways in which technology providers could better support a culturally diverse older population and their families. Social workers attempted to bridge certain gaps, including language line services that did not quite substitute for a native speaker as a first response. Social workers also described how companies need to think beyond language and better understand and represent their audience in outreach materials. Furthermore, both gerotechnology companies and immigrant older adults stand to benefit from culturally sensitive technologies that are targeted specifically to the concerns of older immigrants whose priorities may be more multifaceted than developers and service providers imagine [34]. Certainly, older adults should not be expected or pressured to adopt passive remote monitoring technologies that they feel miss the mark or to continue using technologies that are causing significant negative disruptions to their lives or relationships.

The aim of this analysis was not to predict the various ways in which immigrants from particular countries will respond to home health care technologies. We certainly do not claim that Russian-speaking immigrant elders will uniformly reject passive remote monitoring. Rather, the findings illustrate that the development and implementation of successful interventions require contextualization in social values, which are always culturally inflected. Uniformity of user representations of older adults in technology design and implementation creates barriers to successful use. Indeed, older adults are not a user group but have diverse needs, expectations, and reactions to technologies intended to support them and their caregivers [34-39]. Moreover, the findings highlight why the impact of a particular technology is not fully predictable and should not be imposed on top of existing care practices, but rather, developed alongside these practices [34,40], all of which will benefit from an appreciation for cultural nuance.

### Limitations

This study is exploratory and did not include significant numbers of each user group to make generalizable inferences. Social workers referred to residents using their own grouping terms: “Asian residents and Russian-speaking residents,” 2 groups within which we can expect much cultural variation. Our participants’ own grouping of these residents prevents description of potential cross-country differences. We also note that our findings rely more heavily on reports from social workers regarding cultural differences they observed than on such observations by residents or family members. This is a limitation, and future work with larger sample sizes might examine older adults’ assessments of cultural aspects of remote monitoring technologies in greater depth.

### Conclusions

This study contributes to our understanding of culturally inflected experiences with gerontechnologies and provides insights into its discontinuation for immigrant older adults. Social workers served as the cultural brokers of passive monitoring, and their relationships with immigrant older adults was were informed through their unique immigrant experiences. A more complete understanding of how users, potential users, and discontinued users interact with and experience remote monitoring technology requires an appreciation of the sociocultural context in which these technologies are introduced. This need is more urgent with the growing older population that includes a growing older immigrant population. Successful adoption of gerotechnologies by immigrant older populations could potentially lead to lower costs of long-term care and overall improved quality of life, but only if they can be culturally and practically relevant to these populations. The contextual lens we offer allows us a deeper understanding of the disparate rates of adoption and discontinuation of a sensor-based passive remote monitoring technology intended to help older adults live independently longer.

## Abbreviations

EMS: emergency medical service
PERS: personal emergency response systems

## References

[1] Redfoot D, Feinberg L, Houser A (2013). American Association of Retired Persons.

[2] Ghosh R, Ratan S, Lindeman D, Steinmetz V (2014). Health literacy center.

[3] Cahill E, Lewis LM, Barg FK, Bogner HR (2009). "You don't want to burden them": older adults' views on family involvement in care. J Fam Nurs.

[4] Peine A, Neven L (2019). From intervention to co-constitution: new directions in theorizing about aging and technology. Gerontologist.

[5] Brown N, Webster A (2004). New Medical Technologies and Society: Reordering Life.

[6] Lehoux P (2008). The duality of health technology in chronic illness: how designers envision our future. Chronic Illn.

[7] Neven L (2015). By any means? Questioning the link between gerontechnological innovation and older people's wish to live at home. Technol Forecast Soc Change.

[8] Lehoux P (2006). The Problem of Health Technology: Policy Implications for Modern Health Care Systems.

[9] Cornman JC, Freedman VA (2008). Racial and ethnic disparities in mobility device use in late life. J Gerontol B Psychol Sci Soc Sc.

[10] Resnik L, Allen S (2006). Racial and ethnic differences in use of assistive devices for mobility: effect modification by age. J Aging Health.

[11] Rubin RM, White‐Means SI (2001). Race, disability and assistive devices: sociodemographics or discrimination. Int J of Social Economics.

[12] Gell NM, Wallace RB, LaCroix AZ, Mroz TM, Patel KV (2015). Mobility device use among older adults and incidence of falls and worry about falling: findings from the 2011-2012 National Health and Aging Trends Study. J Am Geriatr Soc.

[13] Ripat J, Woodgate R (2010). The intersection of culture, disability and assistive technology. Disabil Rehabil Assist Technol.

[14] Kaye HS, Yeager P, Reed M (2008). Disparities in usage of assistive technology among people with disabilities. Assistive Technology.

[15] Leidner DE, Kayworth T (2006). Review: a review of culture in information systems research: toward a theory of information technology culture conflict. MIS Quarterly.

[16] Chung J, Thompson H, Joe J, Hall A, Demiris G (2017). Examining Korean and Korean American older adults' perceived acceptability of home-based monitoring technologies in the context of culture. Inform Health Soc Care.

[17] Lutz P (2015). Multivalent moves in senior home care: from surveillance to care-valence. Anthropol Aging.

[18] Population Reference Bureau (2013). Population Reference Bureau.

[19] (2011). United States Census Bureau.

[20] Wong ST, Yoo GJ, Stewart AL (2006). The changing meaning of family support among older Chinese and Korean immigrants. J Gerontol B Psychol Sci Soc Sci.

[21] Hurh W, Kim KC (1990). Religious participation of Korean immigrants in the United States. J Sci Study Relig.

[22] Liem R, Lim BA, Liem JH (2000). Acculturation and emotion among Asian Americans. Cultur Divers Ethnic Minor Psychol.

[23] Willgerodt MA, Miller AM, McElmurry BJ (2002). Becoming bicultural: Chinese American women and their development. Health Care Women Int.

[24] Park M, Chesla C (2007). Revisiting Confucianism as a conceptual framework for Asian family study. J Fam Nurs.

[25] Mui AC (1996). Depression among elderly Chinese immigrants: an exploratory study. Soc Work.

[26] Diwan S, Lee SE, Sen S (2011). Expectations of filial obligation and their impact on preferences for future living arrangements of middle-aged and older Asian Indian immigrants. J Cross Cult Gerontol.

[27] Mackinnon M, Gien L, Durst D (1996). Chinese elders speak out: implications for caregivers. Clin Nurs Res.

[28] Mutchler J, Angel J (2000). Policy development and the older Latino population in the 21st century. J Aging Soc Policy.

[29] Hwang KK (1999). Filial piety and loyalty: two types of social identification in Confucianism. Asian J Soc Psychol.

[30] Corbin J, Strauss A (2008). Basics of Qualitative Research: Techniques and Procedures for Developing Grounded Theory.

[31] Glaser B, Strauss A (1967). The Discovery of Grounded Theory: Strategies for Qualitative Research.

[32] Berridge C (2016). Breathing room in monitored space: the impact of passive monitoring technology on privacy in independent living. Gerontologist.

[33] Kaufman S (2005). And a Time to Die: How American Hospitals Shape the End of Life.

[34] Greenhalgh T, Procter R, Wherton J, Sugarhood P, Hinder S, Rouncefield M (2015). What is quality in assisted living technology? The ARCHIVE framework for effective telehealth and telecare services. BMC Medicine.

[35] Berridge C (2017). Active subjects of passive monitoring: responses to a passive monitoring system in low-income independent living. Ageing Soc.

[36] Dinesen B, Nonnecke B, Lindeman D, Toft E, Kidholm K, Jethwani K, Young HM, Spindler H, Oestergaard CU, Southard JA, Gutierrez M, Anderson N, Albert NM, Han JJ, Nesbitt T (2016). Personalized telehealth in the future: a global research agenda. J Med Internet Res.

[37] (2016). IBM.

[38] Mynatt E, Borrelli A, Czaja S, Iturriago E, Kaye J, Nilsen W, Siewiorek D, Stankovic J (2015). Computing Community Consortium.

[39] Wherton J, Sugarhood P, Procter R, Hinder S, Greenhalgh T (2015). Co-production in practice: how people with assisted living needs can help design and evolve technologies and services. Implement Sci.

[40] Pols J (2017). Good relations with technology: empirical ethics and aesthetics in care. Nurs Philos.

